# Visually integrating and exploring high throughput Phenome-Wide Association Study (PheWAS) results using PheWAS-View

**DOI:** 10.1186/1756-0381-5-5

**Published:** 2012-06-08

**Authors:** Sarah A Pendergrass, Scott M Dudek, Dana C Crawford, Marylyn D Ritchie

**Affiliations:** 1 Center for Systems Genomics, Department of Biochemistry and Molecular Biology, The Pennsylvania State University, Eberly College of Science, The Huck Institutes of the Life Sciences, University Park, PA, USA; 2 Center for Human Genetics Research, Vanderbilt University Medical Center, Nashville, TN, USA; 3 Department of Molecular Physiology & Biophysics, Vanderbilt University Medical Center, Nashville, TN, USA

**Keywords:** PheWAS, Phenome-Wide Association Study, Visualization

## Abstract

**Background:**

Phenome-Wide Association Studies (PheWAS) can be used to investigate the association between single nucleotide polymorphisms (SNPs) and a wide spectrum of phenotypes. This is a complementary approach to Genome Wide Association studies (GWAS) that calculate the association between hundreds of thousands of SNPs and one or a limited range of phenotypes. The extensive exploration of the association between phenotypic structure and genotypic variation through PheWAS produces a set of complex and comprehensive results. Integral to fully inspecting, analysing, and interpreting PheWAS results is visualization of the data.

**Results:**

We have developed the software PheWAS-View for visually integrating PheWAS results, including information about the SNPs, relevant genes, phenotypes, and the interrelationships between phenotypes, that exist in PheWAS. As a result both the fine grain detail as well as the larger trends that exist within PheWAS results can be elucidated.

**Conclusions:**

PheWAS can be used to discover novel relationships between SNPs, phenotypes, and networks of interrelated phenotypes; identify pleiotropy; provide novel mechanistic insights; and foster hypothesis generation – and these results can be both explored and presented with PheWAS-View. PheWAS-View is freely available for non-commercial research institutions, for full details see http://ritchielab.psu.edu/ritchielab/software.

## Background

In Phenome-Wide Association Studies (PheWAS), the association between single nucleotide polymorphisms (SNPs) and an extensive range of phenotypic measurements are calculated in a high throughput, unbiased manner. The phenotypic data used in PheWAS can come from a variety of sources. One possible source is epidemiologic health surveys linked to genotypic data that include measurements of intermediate traits or biomarkers such as blood cell counts and blood pressure measurements, as well as information on case/control status for multiple clinical conditions and risk factors such as presence/absence of diabetes or hypertension. One such example is the Population Architecture Using Genomics (PAGE) network, which is a National Human Genome Research Institute (NHGRI)-supported network of four study sites and a coordinating center accessing eight extensively characterized studies for PheWAS studies in diverse populations [1,2]. These survey-based PheWAS efforts are complimentary to on-going PheWAS efforts using electronic medical records linked to biorepositories such as those in the electronic Medical Records & Genomics (eMERGE) network [3,4].

The exploration of data in a PheWAS effort presents several challenges, including the need for data visualization to assist with interpretation of the data. GWA studies of a single or limited number of traits lend themselves to Manhattan plots where p-values for every test of association are plotted by chromosomal location (x-axis) and the level of significance is visualized easily (y-axis). Such a plot does not present the complex relationships that exist between both genotypes and phenotypes in PheWAS. Therefore, to visualize the complex results of PheWAS, we have developed PheWAS-View, software that can be used to create visual summaries of the SNP, gene, phenotype, and association information resulting from these studies. Using specialized tools such PheWAS-View to investigate results on a larger summary level as well as the individual result level is key for interpretation, analysis, and sharing of PheWAS results. While this tool was developed specifically for PheWAS, it could be used in other high throughput bioinformatics data where thousands of association results are being explored.

### Implementation

PheWAS-View was developed in Ruby, using the RMagick graphics library, for use at the command line. Through the use of various options, various output plots are possible. Table 1 shows various commands and optional settings for PheWAS-View.

**Table 1:** **Listed are all the PheWAS-View plotting options, parameters, and flags (format:** ***-flag name*) for creating PheWAS-View plots using phewas_view.rb**

**Usage: phewas_view.rb**	
**Standard PheWAS Plot**	
**-v, --version**	Show PheWAS-View version
**-e** ***phewas file***	PheWAS-View formatted file for input
**-o** ***output name***	Optional output name for the resultant plot
**-t** ***title***	Main title for the plot (enclose in quotes)
**-f** ***image type***	Image format for output (png default). Other options depend on ImageMagick installation.
**-w, --lowres**	Low resolution image (72 dpi)
**-a, --rotate**	Rotate final image 90 degrees
**-p** ***p-value threshold***	p-value threshold, values less significant will be plotted in grey
**-m, --maxp** ***maximum p-value***	Maximum p-value to plot. Values less significant than the specified cut off are not plotted
**-R, --redline** ***p-value***	Draw a red line at the designated p-value
**-b**	Include direction of effect on plot
**-A, --samp-size**	Include sample size plot
**-l** ***ancestry map file***	Optional ancestry map file
**-c** ***phenotype class names***	Only results matching this phenotype class name are plotted
**-B, --showbest**	Display detailed information for best score at each phenotype
**-x** ***phenotype/SNP file***	PheWAS expected SNP/phenotype file
**-r** ***ancestry***	List of race/ethnicities to include (AA, EA, MA)
**-s** ***SNP ID***	SNP ID to display from input file
**-L** ***phenotype list file***	Optional phenotype list for inclusion
**-N, --no-lines**	No background lines drawn on plot
**-C** ***phenotype correlation file***	Optional file with phenotype correlations
**Sun Plot Settings**	
**-S**	Produce sun plot
**-s** ***SNP ID***	SNP ID to display in center of sun plot
**-P** ***phenotype name***	Phenotype to display in center of sun plot
**-g** ***gene name***	Gene to display in center of sun plot
**-G**	Include gene name along with SNP, when SNP is selected for sun plot
**-E**	Include ancestry as description of result for sun plot
**-m, --maxp** ***p-value***	Choose a p-value threshold, p-values less significant will not be plotted
**-p** ***p-value***	For plotted results, any results more significant will be plotted in red
**-b**	To apply direction of effect for phenotypes in sun plot, - is negative direction, + is positive direction

A single input file is required to produce a standard PheWAS-View plot (example Additional file 1). Required columns for the standard input file include a column of unique SNP identifiers such as an rs number, a column of a unique phenotype description/identification for the tests of association that were calculated for each SNP, and a column of p-values for each test of association. By adding additional columns, additional features are possible with PheWAS-View (example Additional file 2). Table 1 lists the various parameters/flag settings available for modifying PheWAS-View plots (format: *-flag name*) at the command line.

## Results and discussion

**Figure 1:**
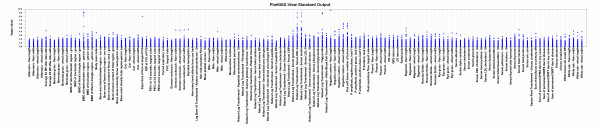
**Standard PheWAS-View Output Example Plot.** A series of simulated PheWAS results plotted in PheWAS-View for a group of phenotypes. The y-axis presents –log_10_(p-value) of the tests of association for all SNPs for each phenotype, and the x-axis represents individual phenotypes. In this way all results for all SNPs are plotted for each phenotype.

One way to inspect initial PheWAS results is first through visualizing all association results across phenotypes using PheWAS-View. Figure 1 shows simulated PheWAS results plotted in standard PheWAS-View format for a series of phenotypes, using the example Additional file 1. The plot is similar in style to a Manhattan plot, where the y-axis represents the magnitude of the association results in –log_10_ (p-value). However, unlike a Manhattan plot where the x-axis represents genetic location, the x-axis of a PheWAS-View plot represents each phenotype from the tests of association, plotted in the order phenotypes are listed in the PheWAS-View input file. This file can be sorted in a number of ways such as by ascending or descending p-value, and then plotted in PheWAS-View, (such as Additional file 3: Figure S1) to aid in exploration of the results.

**Figure 2:**
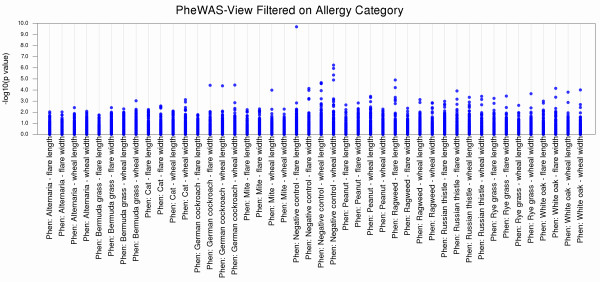
**Selecting Phenotypes for the Plot.** PheWAS-View allows the user to filter the data on various criteria, including limiting the plot to a specific group or list of phenotypes. This is an example of filtering on the phenotype class “Allergy” in PheWAS-View. The data plotted only include those phenotypes that were notated as being in the allergy phenotype class. PheWAS-View has an option for supplying two pieces of phenotypic information, a short and a long phenotype description, in this case the short phenotypic description for all results is “Phen”.

PheWAS-View options allow for modified views of results that may aid in further result investigation and interpretation. For instance, with an extensive number of phenotypes, the plot may become very large. One option for managing plot size is using a filter based on a phenotype group or class. Phenotypes that are related can be given a unique group identifier. If this information is supplied in a column titled “phenotype_class”, the PheWAS-View output can be filtered on any phenotype class of choice (using command line parameter -c *phenotype class*). In this way, only results for phenotypes within that phenotype class are plotted. Figure 2 shows an example filtering on the phenotype class “Allergy” in PheWAS-View using Additional file 2, thus limiting all the plotted results to allergy related phenotypes. An alternate approach is to use the –L flag, and supply a list of phenotypes to filter the resultant PheWAS-View plot for, whereby the results for only those specific phenotypes are plotted. Instead of (or in addition to) filtering results by phenotype group, data can also be filtered by SNP rsID using the parameter -s *SNP ID*.

**Figure 3:**
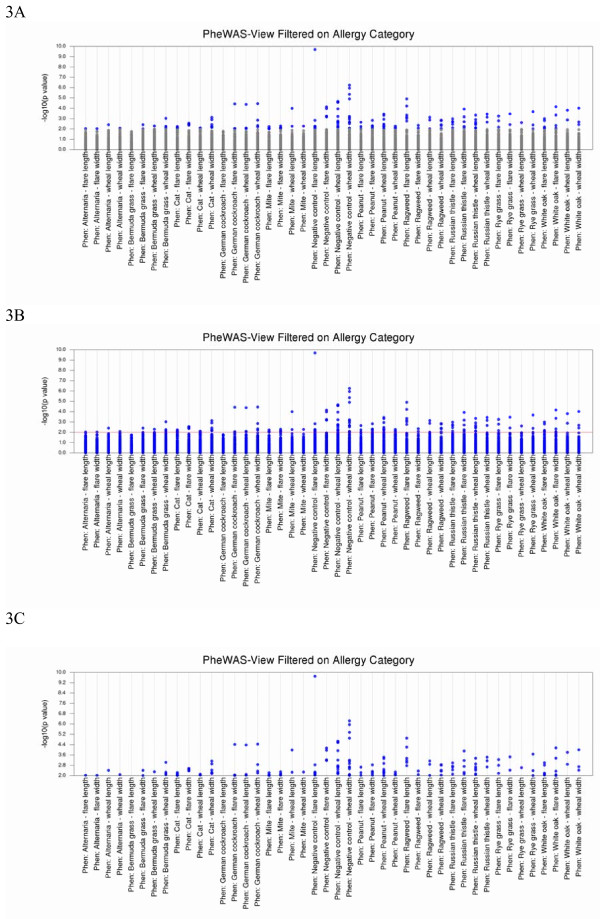
**Options for Highlighting Results Based on p-value Thresholds.** Figure 3A shows the result of using the parameter -p *p-value*, where results more significant than a specified p-value threshold are plotted in blue (in this case p = 0.01), and the other results are plotted in grey. Figure 3B shows the result of using the parameter -R *p-value* to plot a red line at a p-value of interest (p = 0.01). Figure 3C shows the results of using the parameter –m *p-value* to plot only those values more significant than a chosen p-value threshold (p = 0.01).

Multiple PheWAS-View options exist for highlighting results more significant than a specific p-value threshold, which may highlight results of interest. Using the parameter -p *p-value*, results that are more significant than a specific threshold are plotted in blue, and the other results are plotted in grey (Figure 3A). Alternately, a red line can be applied at a p-value of interest (−R *p-value*) (Figure 3B), or through using (−m *p-value*) to plot only results more significant than a p-value threshold (Figure 3C).

**Figure 4:**
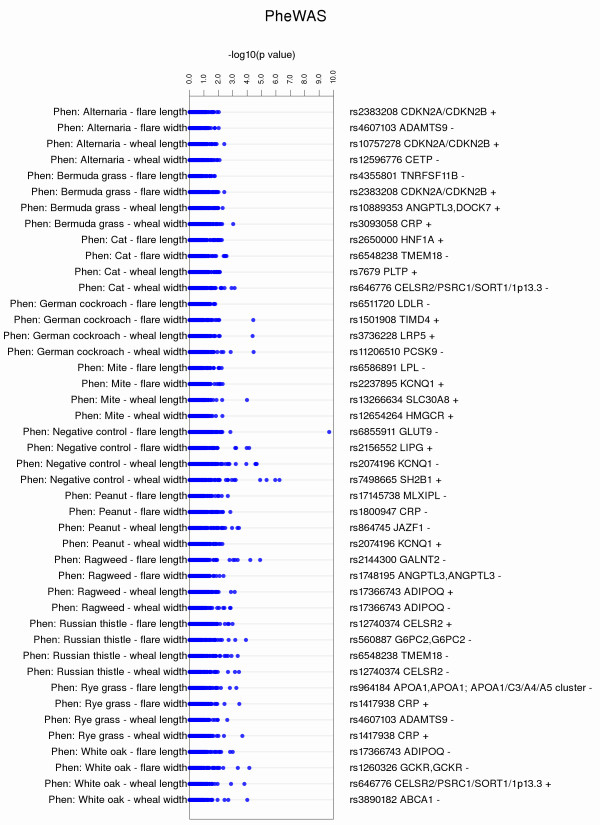
**Vertical Format for PheWAS-View Plots.** PheWAS-View allows results to be plotted in a vertical format. Figure 4 shows the same data used in Figure 2, plotted in a vertical format through using the parameter –a. Compared to previous figures in this manuscript, the phenotypes are now listed along the y-axis, and the x-axis represents –log_10_(p-value) of the tests of association. In addition, using the parameter –B will plot the SNP identifier as well as direction of genetic effect for the association (+ for positive direction of effect, - for negative) for the most significant association for each phenotype listed. Plotting PheWAS results this way facilitates the reading of phenotype descriptions, while still allowing the most significant SNP-phenotype results to be inspected visually.

A useful alternate view is to plot the same information in a vertical format (−a), where the phenotypes are listed along the y-axis, and the x-axis are the –log_10_(p-value) from the tests of association (Figure 4). In this format, reading the phenotype identification is easier, while the most significant SNP-phenotype results can still be identified visually.

For plots in vertical format, using the parameter –B will plot the SNP identifier, gene symbol, as well as direction of the genetic effect (positive (+) or negative (−)) for the most significant p-value for each phenotype (Figure 4). To plot effect size, these data must be provided in a column “ES”, and gene symbol must be provided in a column “Gene” (using example file Additional file 2).

**Figure 5:**
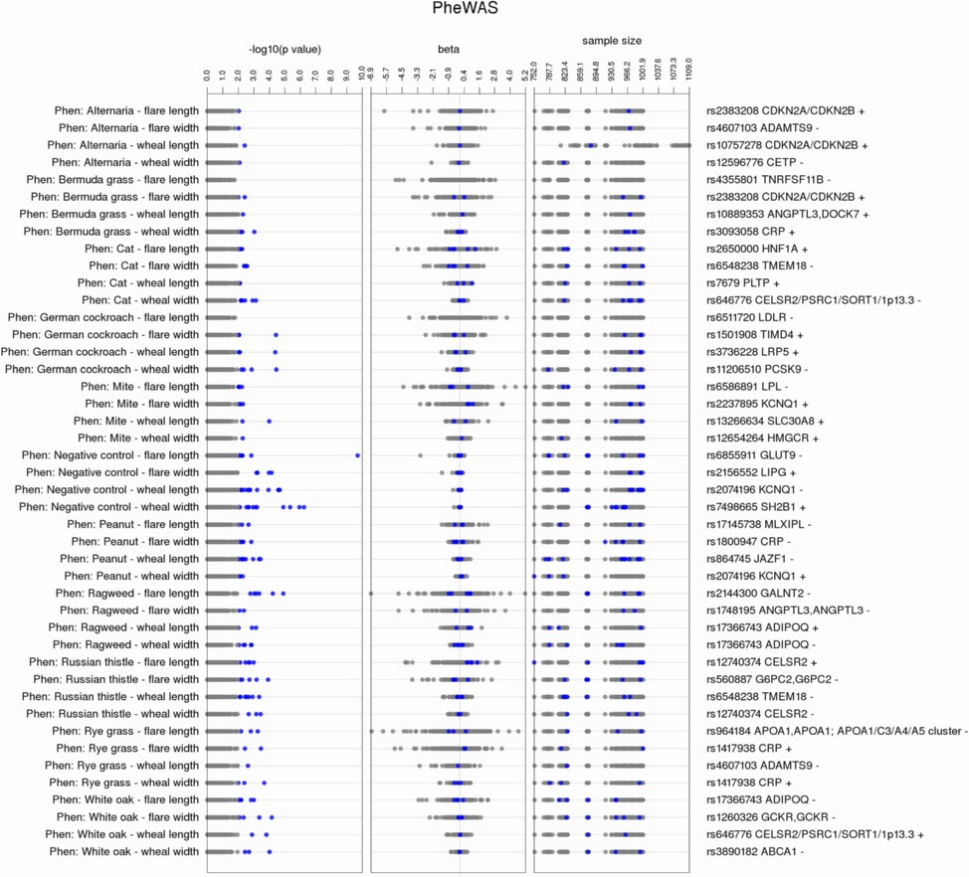
**Additional Information on Sample Size and Direction of Effect.** For this plot the –b parameter was used to plot the direction of effect for all associations for each phenotype in a separate track. The sample size for each association was also plotted in a separate track using the –A parameter.

Figure 5 shows an example plotting the magnitude of the effect size track in vertical format using the -b parameter, as well as plotting the sample size for all tests of association for each phenotype using the –A parameter, with only points passing a p-value < 0.01 in blue.

**Figure 6:**
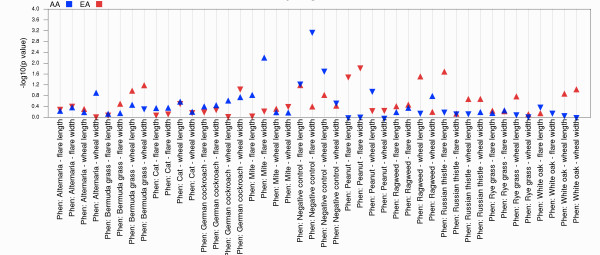
**Comparisons Across Groups.** If the PheWAS analyses are stratified across multiple genetic ancestries or groups, it can be useful to view the similarities and differences of the significance of an association and direction of effect across groups. The output plot can be filtered by a single SNP (using –s SNP rs ID) and one or more groups (−r group1, group2, …) by specifying an identifier for specific results in a column labeled “Groups” in the input file (example in Figure 6). Triangles point up for direction of effect that is positive, and point down for direction of effect that is negative. A different color represents each group.

If the PheWAS analyses are stratified by population or genetic ancestry or other grouping, it can be useful to view the similarities or differences in the significance of an association and direction of effect across groups. The output plot can be filtered by a single SNP (using –s *SNP rs ID*) and one or more groups (−r *group1, group2, …*) by specifying an identifier for specific results in a column labeled “Groups” in the input file. Figure 6 shows results for the SNP rs673548, and African Americans (AA) and European Americans (EA), filtered by just “Allergy” phenotypes. Each group is represented by a different color, and triangles point up for direction of genetic effect that is positive, and point down for direction of genetic effect that is negative. PheWAS-View recognizes the population abbreviations listed in Table 2 and used in 2 2. To use other populations or genetic ancestry or group abbreviations, an alternate group map file can be supplied by the user (−l *filename.txt*) (example Additional file 4).

**Table 2:** PheWAS-View recognizes the population or genetic ancestry abbreviations listed below that can be used as “group” identifiers in the input file

**Label**	**Color**	**Description**
**EA**	red	European American
**AA**	blue	African American
**H**	green	Hawaiian
**API**	purple	Asian Pacific Islander
**AI**	orange	American Indian

**Figure 7:**
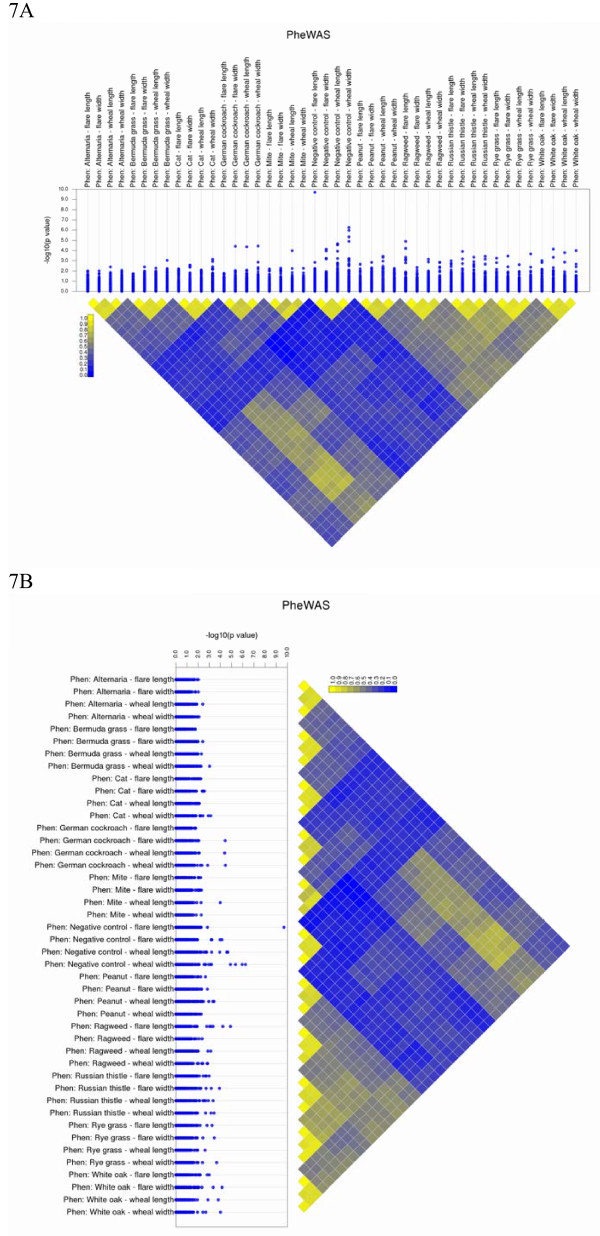
**Phenotypic Correlation Heatmap.** A heatmap of correlations can be plotted with PheWAS-View if a matrix of all pairwise correlation coefficients between phenotypes is plotted. Figure 7A is a PheWAS-View plot with the addition of a correlation heatmap. The cells of the correlation plot range from yellow to blue in the direction of decreasing absolute value of the correlations. Figure 7B shows the same plot in vertical rather than horizontal format after using the –a parameter.

Within PheWAS, significant phenotype-genotype results between SNPs may be due more to the relationship between phenotypes rather than the independent associations between a genetic variant and multiple phenotypes (known as pleiotropy). For instance, if a series of cardiovascular disease-related measurements are highly correlated, the resultant SNP-phenotype associations may be very similar between all the cardiac disease phenotypes due to the correlation between the phenotypes. PheWAS-View can help distinguish between correlated phenotypes and possible pleiotropy. If pairwise phenotype correlations are exhaustively calculated and saved in tab-delimited matrix form, the absolute value of the correlation coefficients can be plotted using PheWAS-View by using the -C *correlation file*. Figure 7A is an example PheWAS-View plot with the phenotypic correlation heat map, Figure 7B shows the same plot in vertical rather than horizontal format, where the cells of the correlation plot range from yellow to blue in the direction of decreasing absolute value of the correlations. Additional file 5 is the example correlation matrix used for Figure 7A and B.

**Figure 8:**
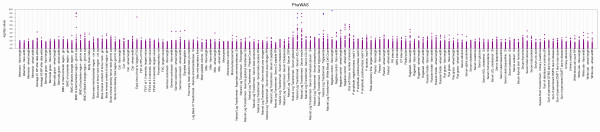
**Distinguishing “Expected” and “Novel” Associations Using Color.** In this plot, novel associations with p < 0.01 are plotted in a different color (purple) from results that are more expected (blue). Using color to distinguish expected and more novel results facilitates prioritizing results to investigate further

Results can also be plotted with “expected” association results in blue, and novel associations plotted in purple by supplying a file of SNPs matched to individual phenotypes that are expected results (−x *phenotype/SNP file*), Figure 8, using Additional file 1 and Additional file 6). Decisions about expected versus unexpected results are a study-by-study decision of the researchers involved. One example would be to consider previously reported SNP-phenotype associations as expected and previously unreported SNP-phenotype associations as novel. Plotting the results with two different colors can be extremely useful to contrast results considered novel and those due to known genotype-phenotype relationships.

**Figure 9:**
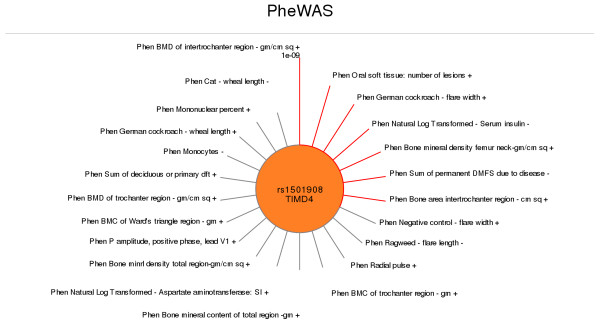
**Sun Plot of Association Results for a Single SNP.** PheWAS results plotted for a single SNP with p < 0.05 (−m p-value). The length of the line corresponds to the significance of the p-value, with the most significant result at the top (“noon”) sweeping around clockwise. The p-value of the most significant result is listed to provide a sense of scale. The lines are red for p-values more significant than a threshold of p = 1x10^-3^ (−p p-value). To add a “-” or “+” for direction of genetic effect for each phenotype use the flag –b.

PheWAS also provides an alternate way to visualize all significant results for a single SNP or phenotype through a “sun plot”. Figure 9 shows a sun plot of all PheWAS results plotted for a single SNP with a p-value < 0.05 (using -m *p-value* to set the figure p-value cutoff). The length of the line corresponds to the significance of the p-value, where the more significant the p-value, the longer the line. The most significant result is at the top of the plot, with the p-value of the most significant result listed to provide a sense of scale. The remaining results sweep around clockwise. The lines are red for p-values more significant than a threshold of p = 1x10^-3^ (using -p *p-value* to specify a threshold for lines being red or grey). To create this figure using Additional file 2, additional parameters were used: -S to create the sun plot, -s with SNPID to identify the specific SNP of interest for the sun plot, and –g to add the gene symbol to the plot. To add a “-” or “+” for direction of effect for each association test, the -b flag was used with the Additional file 2, which contains direction of genetic effect information. If there is genetic ancestry or population information, using the -E flag will indicate the group for each of the associations. Other options for sun plots include plotting all results for a single gene at a p-value threshold (using –g *gene name*), or a single phenotype at a p-value threshold (using –P *phenotype name*) (not shown here).

### Conclusions

The PheWAS approach provides a way to explore pleiotropy and the interrelationships between phenotypes, and as well as generate new hypotheses about the genetic architecture of complex traits. Visualizing complex PheWAS results with the various possible plots available within PheWAS-View provides a way to explore the data in a visual way, facilitating data analysis and interpretation. This software could be also be used for other phenotypically rich association studies such as expression quantitative trait loci (eQTL) studies, studies that have high numbers of phenotypes due to the expression of multiple genes coupled with genotypic data.

### Availability and requirements

**Project name:** PheWAS-View

**Project home page: **http://ritchielab.psu.edu/ritchielab/software

**Operating systems(s):** Linux, Mac OS X, Windows

**Programming language:** Ruby

**Other requirements:** RMagick

**License:** GNU General Public License

**Any restrictions to use by non-academics:** The use of PheWAS-View is restricted to academic and non-profit users

### Competing interests

The authors declare that they have no competing interests.

### Authors’ contributions

SAP carried out design of PheWAS-View, SMD was the software developer. DCC and MDR participated in design, coordination, and drafting of the manuscript. This work was funded by the following grants: LM010040, HG004798, and U01HG004798. All authors read and approved the final manuscript.

## Supplementary Material

Additional file 1 **The single input file used to produce the standard PheWAS-View plots of Figure **1**, Figure **8**, and Additional file **3**.** Click here for file

Additional file 2 **The input file used to produce the PheWAS-View plots of Figures **3,4**,**5,6**,**7**, and **9**.** Adding additional information columns allows for the use of additional features within PheWAS-View. Click here for file

Additional file 3 **Figure S1 Standard PheWAS-View Output Example Plot Sorted by P-value.** A series of simulated PheWAS results plotted in PheWAS-View for a group of phenotypes. The y-axis presents –log_10_(p-value) of the tests of association for all SNPs for each phenotype, and the x-axis represents individual phenotypes. In this way all results for all SNPs are plotted for each phenotype. This plot was created using the same data as Figure 1, plotted sorting the input data file by p-value. Click here for file

Additional file 4 This is an example of the type of file that can be supplied by the user in order to use other population, genetic ancestry, or group abbreviations. Click here for file

Additional file 5 **This is the example phenotype correlation matrix input file used for Figure **7A**and Figure **7B**.** Click here for file

Additional file 6 **The example file used to plot “expected” association results in blue, and novel associations in purple for Figure **8**.** Click here for file
