# Supplementary material for: Visually integrating and exploring high throughput Phenome-Wide Association Study (PheWAS) results using PheWAS-View
Source: BioData Min. 2012 Jun 8;5:5. doi: 10.1186/1756-0381-5-5 (PMC3476448; doi:10.1186/1756-0381-5-5)
Supplement: Additional file 3 — Figure S1 Standard PheWAS-View Output Example Plot Sorted by P-value. A series of simulated PheWAS results plotted in PheWAS-View for a group of phenotypes. The y-axis presents –log10(p-value) of the tests of association for all SNPs for each phenotype, and the x-axis represents individual phenotypes. In this way all results for all SNPs are plotted for each phenotype. This plot was created using the same data as Figure 1, plotted sorting the input data file by p-value. [file 1756-0381-5-5-S3.doc]

PheWAS-View


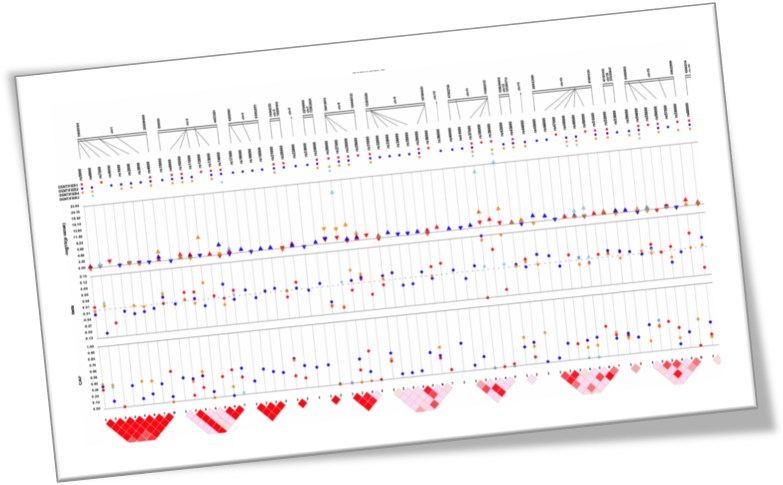


Visualization and interpretation of

Phenome-Wide Association Study (PheWAS) Results

Table of Contents

Introduction [1](#__RefHeading___Toc191282617)

Installation for Command Line Version [1](#__RefHeading___Toc191282619)

PheWAS-View Command Line Options [4](#__RefHeading___Toc191282620)

Creating a Sample PheWAS-View Plot at the Command Line [4](#__RefHeading___Toc191282621)

PheWAS-View Input File Format [6](#__RefHeading___Toc191282622)

*Standard PheWAS View Plot* [6](#__RefHeading___Toc191282623)

# Introduction

# We have developed the software PheWAS-View for visually integrating PheWAS results, including information about the SNPs, relevant genes, phenotypes, and the interrelationships between phenotypes, that exist in PheWAS. As a result both the fine grain detail as well as the larger trends that exist within PheWAS results can be elucidated.

# Installation for Command Line Version

*For Linux*

*Installing Ruby and ImageMagick are necessary for running PheWAS-View.* A script (ruby_install.sh) is available for loading Ruby and ImageMagick at http://ritchielab.psu.edu/ritchielab/softwarealong with the script for running PheWAS-View.

***To install Ruby and ImageMagick not using the Ruby/ImageMagick installer script:***

1. Download and install Ruby. See the following site for instructions on Linux and OS X.

**http://www.ruby-lang.org/en/downloads/**

2. Download and install ImageMagick. See the following site for instructions on Linux and OS X.

**http://rmagick.rubyforge.org/install2-linux.html**

**http://rmagick.rubyforge.org/install-osx.html**

3. Download phewas_view.rb from

**http://ritchielab.psu.edu/ritchielab/software**

*For Windows*

1. Download and install Ruby. Use the one-click installer as the simplest option.

**http://rubyforge.org/frs/download.php/47082/ruby186-27_rc2.exe**

Enable RubyGems needs to be checked. Otherwise use defaults for installation.

2. Download RMagick windows gem from the rmagick-win32 section of

**http://rubyforge.org/frs/?group_id=12&release_id=35951**

3. Extract to a temporary directory

4. Run **ImageMagick-6.5.6-8-Q8-windows-dll.exe** to install ImageMagick. Accept the default options.

5. Make sure RubyGems is up-to-date. If you are using an older version, update it now. Enter the command

gem update --system

**6. Installing the gem is very simple. Start a Command Prompt window. Use the cd command to make the temporary directory that contains the unzipped files the current directory. Enter this command:

gem install rmagick --local

You should see:

Successfully installed rmagick-2.12.0-x86-mswin32

If instead you get the message

Error installing gem rmagick[.gem]: buffer error

then you didn't update RubyGems. Do so now and re-run the gem install command.

7. Download phewas_view.rb from

http://ritchielab.psu.edu/ritchielab/software

# PheWAS-View Command Line Options

Here are the command-line arguments for controlling the behavior of the PheWAS-View script. All parameters/flags (format: *-flag name*) for creating PheWAS-View plots using phwas_view.rb. Only the –e option is required, specifying the main input for the script to generate the standard PheWAS-View plot.

| **Usage: phewas_view.rb** |  | |
| --- | --- | --- |
| **Standard PheWAS Plot** | |  |
| **-v, --version** | | Show PheWAS-View version |
| **-e *phewas file*** | | PheWAS-View formatted file for input |
| **-o *output name*** | | Optional output name for the resultant plot |
| **-t *title*** | | Main title for the plot (enclose in quotes) |
| **-f *image type*** | | Image format for output (png default). Other options depend on ImageMagick installation. |
| **-w, --lowres** | | Low resolution image (72 dpi) |
| **-a, --rotate** | | Rotate final image 90 degrees |
| **-p *p-value threshold*** | | p value threshold, values less significant will be plotted in grey |
| **-m, --maxp *maximum p-value*** | | Maximum p value to plot. Values less significant than the specified cut off are not plotted |
| **-R, --redline *p-value*** | | Draw a red line at the designated p value |
| **-b** | | Include direction of effect on plot |
| **-A, --samp-size** | | Include sample size plot |
| **-l *ancestry map file*** | | Optional ancestry map file |
| **-c *phenotype class names*** | | Only results matching this phenotype class name are plotted |
| **-B, --showbest** | | Display detailed information for best score at each phenotype |
| **-x *phenotype file*** | | PheWAS expected phenotypes file |
| **-r *ancestry*** | | List of race/ethnicities to include (AA, EA, MA) |
| **-s *SNP ID*** | | SNP ID to display from input file |
| **-L *phenotype list file*** | | Optional phenotype list for inclusion |
| **-N, --no-lines** | | No background lines drawn on plot |
| **-C *phenotype correlation file*** | | Optional file with phenotype correlations |
|  | |  |
| **Sun Plot Settings** | |  |
| **-S** | | Produce sun plot |
| **-s *SNP ID*** | | SNP ID to display in center of sun plot |
| **-P *phenotype name*** | | Phenotype to display in center of sun plot |
| **-g *gene name*** | | Gene to display in center of sun plot |
| **-G** | | Include gene name along with SNP, when SNP is selected for sun plot |
| **-E** | | Include ancestry as description of result for sun plot |
| **-m, --maxp *p-value*** | | Choose a p value threshold, p values less significant will not be plotted |
| **-p *p-value*** | | For plotted results, any results more significant will be plotted in red |
| **-b** | | To apply direction of effect for phenotypes in sun plot, - is negative direction, + is positive direction |

Table 2. PheWAS-View ancestry abbreviations

PheWAS output plots can be filtered by one or more groups (-r group1, group2, …). PheWAS-View recognizes the population or genetic ancestry abbreviations listed below that can be used as “group” identifiers in the input file.

| **Label** | **Color** | **Description** |
| --- | --- | --- |
| **EA** | red | European American |
| **AA** | blue | African American |
| **H** | green | Hawaiian |
| **API** | purple | Asian Pacific Islander |
| **AI** | orange | American Indian |

Creating a Sample PheWAS-View Plot at the Command Line

Type at the command prompt:

**ruby phewas_view.rb –e sample_input.txt**

To specify a title and output file name for the plot:

**ruby phewas_view.rb –e sample_input.txt –t “My sample plot” –o sample**

**For more details and usage of other options see the manuscript:**

**Visually Integrating and Exploring High Throughput Phenome-Wide Association (PheWAS) Results Using PheWAS-View**
